# Barriers to accessing care for cardiometabolic disorders in Malawi: partners as a source of resilience for people living with HIV

**DOI:** 10.1186/s12939-024-02181-9

**Published:** 2024-04-27

**Authors:** Everlyne G. Ogugu, Julie T. Bidwell, Allison Ruark, Rita M. Butterfield, Sheri D. Weiser, Torsten B. Neilands, Nancy Mulauzi, Ethel Rambiki, James Mkandawire, Amy A. Conroy

**Affiliations:** 1 Betty Irene Moore School of Nursing, University of California Davis, Davis, CA USA; 2 Wheaton College, Biological and Health Sciences, Wheaton, IL USA; 3 Department of Medicine, University of California San Francisco, San Francisco, CA USA; 4 Invest in Knowledge, Zomba, Malawi; 5 Lighthouse Trust, Lilongwe, Malawi; 6 Betty Irene Moore School of Nursing, University of California Davis, 2570 48th Street, Sacramento, CA 95817 USA

## Abstract

**Background:**

People living with HIV (PLWH) are at increased risk of cardiometabolic disorders (CMD). Adequate access to care for both HIV and CMD is crucial to improving health outcomes; however, there is limited research that have examined couples’ experiences accessing such care in resource-constrained settings. We aimed to identify barriers to accessing CMD care among PLWH in Malawi and the role of partners in mitigating these barriers.

**Methods:**

We conducted a qualitative investigation of barriers to CMD care among 25 couples in Malawi. Couples were eligible if at least one partner was living with HIV and had hypertension or diabetes (i.e., the index patient). Index patients were recruited from HIV care clinics in the Zomba district, and their partners were enrolled thereafter. Interviews were conducted separately with both partners to determine barriers to CMD care access and how partners were involved in care.

**Results:**

Participants framed their experiences with CMD care by making comparisons to HIV treatment, which was free and consistently available. The main barriers to accessing CMD care included shortage of medications, cost of tests and treatments, high cost of transportation to health facilities, lengthy wait times at health facilities, faulty or unavailable medical equipment and supplies, inadequate monitoring of patients’ health conditions, some cultural beliefs about causes of illness, use of herbal therapies as an alternative to prescribed medicine, and inadequate knowledge about CMD treatments. Partners provided support through decision-making on accessing medical care, assisting partners in navigating the healthcare system, and providing financial assistance with transportation and treatment expenses. Partners also helped manage care for CMD, including communicating health information to their partners, providing appointment reminders, supporting medication adherence, and supporting recommended lifestyle behaviors.

**Conclusions:**

Couples identified many barriers to CMD care access, which were perceived as greater challenges than HIV care. Partners provided critical forms of support in navigating these barriers. With the rise of CMD among PLWH, improving access to CMD care should be prioritized, using lessons learned from HIV and integrated care approaches. Partner involvement in CMD care may help mitigate most barriers to CMD care.

## Background

By the end of 2022, an estimated 39 million people were living with HIV worldwide, with two-thirds of them residing in Sub-Saharan Africa (SSA) [1]. As the use of antiretroviral therapy (ART) has become more widespread and led to increased life expectancy among people living with HIV (PLWH), noncommunicable conditions, including cardiometabolic disorders (CMD) such as hypertension, diabetes, and cardiovascular disease (CVD), have become more prevalent in this population [2, 3]. Cardiovascular disease has emerged as one of the leading causes of morbidity and mortality among PLWH [4–6]. Results from systematic reviews and meta-analyses reveal a nearly twofold increased risk of CVD among PLWH compared to HIV-negative individuals [7, 8]. This increased risk of CVD can be attributed to various factors, including HIV-associated inflammation and the use of ART, in addition to traditional risk factors such as cigarette smoking, obesity, aging, hypertension, and diabetes [2, 7, 9]. In the last two decades, HIV-associated CVD has tripled globally, with some of the largest burdens of disability-adjusted life-years (DALYs) occurring in SSA [7].

Malawi, currently classified as a low-income economy country [10], is one of the SSA countries with a high burden of HIV and non-communicable diseases. Malawi has a high prevalence of HIV (8.9% of adults) [11], and the prevalence of hypertension and diabetes is estimated to be 16-33% and 2-6%, respectively [12]. Recent studies conducted among PLWH in Malawi have shown that 20–24% and 2-7% of adults receiving HIV treatment have hypertension and diabetes, respectively [9, 13–15], and a substantial number of CMD cases in PLWH remain undiagnosed, thereby limiting access to timely CMD treatment [9, 14]. The control of CMD in those on HIV care is also a growing challenge [13]. Findings from longitudinal studies in Malawi of hypertensive PLWH indicate hypertension control rates are notably low in this population, with only 19-28% attaining blood pressure control [13, 16, 17]. In contrast, Malawi has made substantial progress in HIV care as indicated by the 2023 Joint United Nations Programme on HIV/AIDS (UNAIDS) report showing that 94% of those on ART have achieved HIV viral suppression [18]. The success in HIV care is largely attributable to the investment in and establishment of comprehensive HIV management programs in SSA, including in Malawi, which have enhanced equitable access to HIV care [18, 19]. The success achieved by Malawi in HIV management despite the major economic constraints it faces [20], presents an opportunity to leverage lessons learned from HIV care programs to develop effective strategies for CMD care in resource-constrained settings.

Access to adequate care for both HIV and CMD is crucial in improving and maintaining positive gains achieved in health outcomes for PLWH over the last few decades. Various challenges have been highlighted in accessing CMD care for PLWH in Malawi, including frequent medication shortages in government facilities, health care staff shortages, treatment-related costs, the use of herbal medicine instead of seeking conventional medical treatment, a lack of symptoms and feeling healthy, and inadequate monitoring of patient progress, among others [12, 13, 21, 22]. Promoting equitable access to CMD care is pivotal for mitigating associated disease burden and financial strain on healthcare systems and communities [23].

**Fig. 1:**
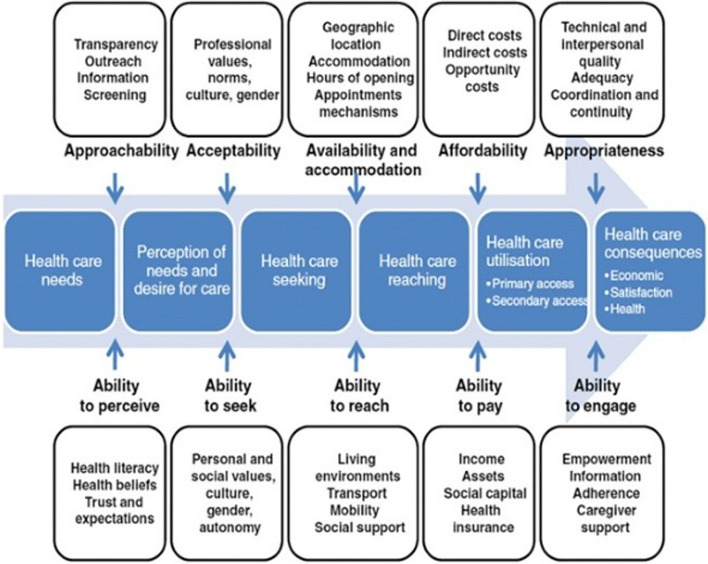
Levesque’s conceptual framework of access to health care reprinted with permission

In their conceptual framework of healthcare access, Levesque et al. define access as “the opportunity to have health care needs fulfilled” [24, p. 4] and outline supply-side (healthcare system) and demand-side (individuals needing care) dimensions of access that interact to determine accessibility to care. The supply-side dimensions include approachability, acceptability, availability and accommodation, affordability, and appropriateness. Corresponding to these dimensions on the demand side are the abilities of individuals needing care, which include the ability to perceive, seek, reach, pay, and engage (Fig. 1) [24]. Levesque et al.’s model has been used previously in Malawi to study access to maternal child health services [25], and in the present study, we used this framework to guide the identification of barriers to accessing CMD care. Our main rationale for selecting this framework is that it provides a comprehensive way of examining determinants of health care access along a continuum, ranging from perceiving the need for health care to achieving the expected outcomes [24].

One important dimension of the Levesque et al. model is the inclusion of social support across the dimensions of the demand side (e.g., caregiver support’s impact the ability to engage), as shown in Fig. 1. The significant role of dyadic couple relationships in the management of chronic health conditions is well established [26, 27], and among married individuals seeking healthcare services, the spouse is commonly named as the primary source of social support [26]. Studies among PLWH from Malawi and South Africa indicate that supportive couple relationships are important for managing chronic disease in settings with limited healthcare resources [28–31]. Previous research in Malawi and other countries has shown that social support by a spouse is associated with positive health outcomes in HIV [32–34], hypertension [35] and diabetes [36]. In Malawi, over two thirds of adults are married and live together [37], and in HIV care programs, partners are often the designated HIV treatment guardian [28, 38]. Therefore, supporting partner involvement in both HIV and CMD care has potential to achieve greater and more sustainable impact in health outcomes in Malawi [39]. Currently, little is known about how partners may help to mitigate barriers to CMD care in resource-constrained settings like Malawi, which could inform whether and how partners could be involved in care.

We sought to fill these gaps by conducting a qualitative study with couples living with HIV and CMD to identify the key barriers to accessing CMD care among persons living with HIV and CMD and to understand the role of partners in helping to mitigate barriers to accessing CMD care. By conceptualizing access to care as a couple-level issue in which partners may offer social support, we extend current literature on patient perspectives of barriers to CMD care by bringing in the perceptions and experiences of both partners together. Expanding this framework to couples will ultimately inform intervention approaches that move beyond the individual patient to the broader social environment in which access to care is embedded.

## Methods

### Sample and recruitment

This study is part of Healthy Hearts, a mixed-methods observational study conducted in Zomba District, Malawi, focusing on couples living with both HIV and CMD (hypertension and diabetes). The qualitative phase of Healthy Hearts aims to explore couples' beliefs, knowledge, and experiences regarding the management of HIV and CMD. Malawi is predominantly rural, with approximately 84% of its population residing in rural areas [40]. Between October and December 2021, twenty-five couples, where at least one partner was diagnosed with both HIV and CMD (the index patient), were recruited for participation. These couples were selected from three outpatient HIV care clinics at three different health facilities across Zomba District: a government health center in a peri-urban area, a tertiary government hospital in Zomba City, and a private rural community hospital.

We used purposive sampling with the goal of recruiting equal numbers of index patients by gender, CMD type, and recruitment site, as well as equal numbers of couples who were discordant and concordant for both HIV and CMD. Eligible couples were age 18 or older (both partners), were married or cohabiting for at least six months, and had disclosed their HIV and CMD status to their spouse. Index patients were recruited in waiting rooms when attending an HIV care appointment and screened for eligibility based on self-reported CMD and HIV status, which was later confirmed against the medical records. Eligible patients were given study information to share with their spouses, who could then contact the research team if interested. In some cases, partners were recruited first when picking up the index patients’ medications, and a similar process was followed. In all the cases, both partners needed to be eligible, be interested in participating, and provide informed consent.

### Data collection

Research assistants trained in qualitative interviewing techniques conducted in-depth interviews in Chichewa (the local language). The interviewers were matched by gender to the participants, and the interviews lasted approximately 90 minutes. Both partners were interviewed separately but simultaneously in private rooms at the HIV clinics to obtain independent perspectives, avoid bias, and ensure confidentiality of responses, particularly for sensitive questions about relationships or HIV. Participants also completed a brief questionnaire on demographic information, relationship characteristics, and clinical variables, such as length of time on ART. The interview guide included open-ended questions on theoretical constructs, including relationship quality, coping with disease, experiences of HIV and CMD health care, and disease management, which were based on Helgeson and colleagues’ communal coping and adjustment to chronic diseases framework [41]. In identifying barriers to accessing health care, we elicited both partners’ perspectives on patients’ experiences with CMD care, including health advice and medications provided, whether there were challenges with accessing care, and if yes, what factors played a role. The interview questions were tailored towards the participant’s disease status (i.e., HIV, hypertension, or diabetes) and whether the participant was the index patient or support partner.

### Data analysis

The interviews were audio-recorded, transcribed, and translated from Chichewa to English. The transcripts were spot-checked for completeness and correctness by a research manager who is fluent in both languages. We analyzed data at the individual and dyadic levels using framework analysis, which involves using data matrices to organize data by themes and cases [42]. In this case, we read through each pair of couple interviews and abstracted the data into matrices organized by couple and by themes derived from the interview guides and the interview data. We also compared within-couple accounts (i.e., between partners), made notes about areas of agreement and disagreement, and wrote a memo for each couple summarizing key details of their accounts and how they represented themselves. The research team held regular meetings to present findings, refine our list of codes, and discuss emerging themes.

After the data were abstracted into matrices, we further analyzed a subset of data with the higher-level codes labeled ‘access to care,’ ‘beliefs about causes, treatment, prevention of HIV and CMD,’ ‘management of HIV and CMD,’ ‘concerns about HIV and CMD,’ ‘use of traditional healer/ herbs,’ ‘shared illness appraisal,’ and ‘partner support.’ We coded for the main categories and sub-categories of barriers to care and by type of disease to identify barriers unique to hypertension and diabetes. The key findings on barriers to CMD care access were organized using Levesque et al.’s conceptual framework of access to health care [24]. Next, we coded for strategies used by index patients and forms of support provided by their partners in mitigating barriers to care. Finally, we quantified the types of barriers reported and types of support offered by partners to overcome access to care issues to explore patterns in the data.

## Results

### Participants’ characteristics

Twenty-five couples (*n*=50 individuals) were enrolled in the study. The median age of the participants was 51 years (range 37 to 66). All couples were married. Most (68%) participants had a primary school education or less. More than three-quarters (80%) of the participants were living with HIV and were on ART. Fifty-four percent of the participants (21 index patients and six partners) had hypertension, whereas 14% (five index patients and two partners) had diabetes. All participants with CMD stated that their spouse was aware of their CMD diagnosis. The median time since hypertension and diabetes diagnosis was 5.5 and 2.8 years, respectively. The majority (85%) of patients with hypertension were on antihypertensive medication, and 86% of patients with diabetes were on antidiabetic medication. Medication adherence varied across all three conditions, with 85%, 65%, and 100% of patients reporting that in a typical week, they never missed taking medications for HIV, hypertension, or diabetes, respectively (Table 1).

**Table 1:** Characteristics of the study participants

	Women (*N* = 25)		Men (*N* = 25)		Total (*N* = 50)	
	% (N)	Median (IQR)	% (N)	Median (IQR)	% (N)	Median (IQR)
Age, years		47 (44, 53)		56 (49, 61)		51 (47, 56)
Relationship duration, years						21 (10, 32)
Education level						
No formal education	24% (6)		8% (2)		16% (8)	
Primary school (Grades 1-8)	60% (15)		44% (11)		52% (26)	
Some high school or higher	16% (4)		48% (12)		32% (16)	
Recruitment site						
Urban tertiary government hospital					56% (28)	
Private rural community hospital					36% (18)	
Peri-urban government health center					8% (4)	
Living with HIV	80% (20)		80% (20)		80% (40)	
Time since diagnosis, years		12 (8, 14)		9 (7, 14)		11 (7, 14)
Currently taking ART	100% (20)		100% (20)		100% (40)	
Never misses a dose of ART in a given week	75% (15)		95% (19)		85% (34)	
Living with HTN	68% (17)		40% (10)		54% (27)	
Time since diagnosis, years		6 (2, 13)		5 (2, 8)		6 (2, 10)
Currently taking HTN medication	100% (17)		60% (6)		85% (23)	
Never misses a dose of HTN medication in a given week	59% (10)		83% (5)		65% (15)	
Living with DM	4% (1)		24% (6)		14% (7)	
Time since diagnosis, years		13		2 (1, 5)		3 (1, 12)
Currently taking DM medication	100% (1)		83% (5)		86% (6)	
Never misses a dose of DM medication in a given week	100% (1)		100% (5)		100% (6)	

*Abbreviations*: *ART* Antiretroviral therapy, *HTN* Hypertension, *DM* Diabetes mellitus,* IQR *Interquartile range

### Barriers to CMD care access

The findings on barriers to accessing CMD care are organized and presented under the five dimensions of Levesque’s conceptual framework of access to health care [24]. In the interview excerpts, the index patient is referred to as the ‘Patient,’ and their spouse is referred to as the ‘Partner.’The excerpts also highlight whether the participant resides in a rural or urban area. 

#### Barriers related to approachability and ability to perceive

This theme focuses on the degree to which the healthcare facilities provide sufficient information about their services to the population needing those services and the ability of the individuals needing CMD care to perceive the need for such care [24].

#### Cultural beliefs about illnesses that may delay access to conventional health care.

A few of the participants with CMD reported seeking help from traditional healers when they first developed symptoms of illness before deciding to visit health facilities when the symptoms worsened. In most instances, the participants reported that they never sought the services of traditional healers again when they received a medical diagnosis.

The only time [when I sought the services of a traditional healer] was before I was diagnosed [with hypertension]. When I had bad headaches, people cooked for me herbal medicine for the headache, and people made cuts on my body. But when I got to the hospital and found out that it was hypertension, I never took medicine from any other person” (Couple 01 – Female patient with HIV and hypertension. Rural resident)

One reason for initially seeking the services of a traditional healer was the attribution of the symptoms one was experiencing to being bewitched. The term “bewitch” is used in some communities to refer to the act of causing harm to someone using supernatural forces [43].

He [the traditional healer] just gave me roots. They did not help me… The sugar levels were rising…. The issue is that, where I am staying, they [the persons who the participant believes caused the bewitchment] wanted to chase us from the place. I saw that they wanted to kill me, that is why I sought help from the traditional healer. Then I saw that the condition was never changing. My body kept being weak (Couple 07 – Male patient with HIV, diabetes, and hypertension. Rural resident)

#### Barriers related to acceptability and ability to seek

This theme relates to social factors that play a role in the extent to which individuals with CMD seek and accept the care provided by the healthcare system [24].

##### The use of herbal therapies as an alternative versus complementary to conventional medicine

About half of those with CMD reported taking various herbs and food items such as hibiscus powder, lemon grass, garlic, guava leaves, and ginger to control blood pressure or blood sugar. Most of these participants stated that they used these items as complementary therapies to the prescribed medicine, and thus, their use did not interfere with their CMD treatment plan.

Even though all participants were aware that treatment for hypertension and diabetes was provided at healthcare facilities and most sought treatment, a few participants with CMD initially opted to try other alternatives, such as herbal medicine, that they deemed more acceptable than the lifelong use of prescription medicine. Some participants reported that local media communications about alternative therapies for CMD influenced their decision-making about where to seek care. For example, some talked about local radio broadcasts that disseminated information about alternative treatments that purportedly cured diseases such as hypertension and diabetes. As illustrated below by one of the participants, even with limited information about the specifics of these alternative therapies, the narratives circulating via local media about numerous people being cured using these alternative therapies appeared to influence their decision not to seek care at a healthcare facility initially.

I wasn’t feeling well in my body. I was not able to work the way I normally do. I said, “I should go to [name of herbal clinic]. They will tell me what it is”. I heard on the radio that many people are getting cured of this disease there, so I rushed there. When I went there, I saw that there were no pills. It was ground herbs, honey, and other things. Some looked like grass. That is when I said, “Ah, I didn’t know these people were herbalists” …. I did not go to the hospital because I wanted first to know if the [herbal] medication would help me (Couple 04 – Male partner with diabetes. Rural resident)

Another participant’s narrative reflected similar concerns surrounding the long-term use of prescription medications to the degree that they believed it caused some people to avoid seeking health care at hospitals entirely.

Many people do not come to the hospital because they say that if you start taking hospital medication, then it is for the rest of your life, and so it is better to be taking natural herbs (Couple 07: Female partner of a patient with HIV, diabetes, and hypertension. Rural resident).

One participant, who was taking prescribed antihypertensive medications, experimented with alternative strategies that some members of her community had suggested would cure hypertension and, hence, remove the need to take medication. However, the participant ultimately chose to continue with conventional medicine because the alternative therapy was unpleasant.

Some people want to stop taking medicine for hypertension. So, they increase [resting] and eating different foods like garlic. So [the blood pressure] reduces…. You will not go to the hospital again. I tried [garlic], but it was disgusting (Couple 05 – Female patient with HIV and hypertension. Rural resident)

##### Concerns about adverse effects of CMD medications

Misinformation about the adverse effects associated with prescription medicine for CMD could also cause anxiety among those with CMD and negatively impact the acceptance of CMD treatments. For example, there were community members who believed that the medications used to treat hypertension were harmful because they caused other diseases. One participant reported being more worried about hypertension than HIV due to some community members claiming that hypertension medications can cause illness.

The disease that worries me a lot is hypertension [when compared to HIV]. If we could have been listening to people…. some people say that medicine for hypertension is not good. Some people say that when you take the medicine, it causes other things. New diseases come into the body… Some lie that it paralyzes organs – (Couple 09 – Female partner with HIV and hypertension. Rural resident).

#### Barriers related to availability and accommodation and ability to reach

This theme relates to the accessibility of healthcare services for individuals needing CMD care, encompassing the healthcare facility, its services, its team members, and factors that influence the ability of those with CMD to physically get to where the healthcare services are located [24].

##### Shortage of CMD medications at government health facilities

The most cited barrier to CMD care was medication shortages at government health facilities, which necessitated purchasing medications at private pharmacies (although not all could afford this). In contrast, ARV therapies were readily available and free of charge at government health facilities. Consequently, many persons living with HIV and CMD had to seek health care from multiple health facilities as mentioned by one of the participants attending a private rural community hospital

The medical care for HIV, we get it for free, but for hypertension, we pay… I go to private clinics, and I pay for the medicine. Why? In government hospitals, they can give you one type of medication, while when you go to private clinics, they give you all three types (Couple 08 - Male patient with HIV and hypertension. Rural resident)

Hypertension medication shortages occurred in health centers as well as at the tertiary health facility as mentioned by a participant whose partner resorted to seeking alternatives to CMD medication, such as the use of herbal therapies, due to medication shortages.

She [index patient] gets medical care for HIV at the health center, but medical care for hypertension is difficult because sometimes she is told that drugs are not available. A while back, she went to [the tertiary government hospital], and I have forgotten what she was told, but she came back home without any drugs. When she failed from all these places, that is when she went looking for herbal medicine to [sort of] be protecting and calming her… So,the main problem is hypertension medicine. It is hard to find (Couple 02 – Male partner of a patient with HIV and hypertension. Rural resident)

However, another participant living with HIV and diabetes stated that they did not experience a problem accessing medications at the tertiary government health facility, which the participant perceived as having a more secure supply of medications.

No treatment is hard for me to get. Maybe because this is a central hospital, I do not know about my friends from smaller clinics…I chose the central hospital because I know that there are a lot of things [drugs and services]. It cannot be possible for drugs to be completely out of stock. That is why I chose this clinic. I have never come here and been told that there are no drugs (Couple 20 – Male patient with HIV and diabetes. Rural resident)

##### Long distance to health facility

To avoid experiencing challenges related to frequent CMD medication shortages, some participants chose to seek care at the tertiary government facility in Zomba City, where CMD medications were less likely to be out of stock. However, for some, these facilities were far from home, leading to other challenges of distance and transport costs.

Where we are from is far, so some people do not have enough money for transport. As you can see, we chose to get treatment from [tertiary government hospital], but it is very far. The money spent on transport could be used to buy maize and do other things. For others to find 1000 Kwacha [about one US dollar], it could take them two weeks, and they will still not be able to find such an amount of money (Couple 20 – Male patient with HIV and diabetes. Rural resident)

One participant who gets healthcare from the tertiary government hospital emphasized the need to have CMD treatment readily accessible within the community to lessen the burden of having to travel long distances, often on foot for hours, to access healthcare services.

There should not be a long distance for us to access medicine. Medicine should be closer to where we are residing. The medication should be available in our local health centers…because with this issue of walking over long distances …some people fail to come [get medication] because maybe they are lacking transport to catch a bus (Couple 19 – Male patient with HIV and hypertension. Urban resident)

#### Barriers related to affordability and ability to pay

This theme concerns the financial capability of individuals to access the healthcare services they need [24].

##### Cost of CMD treatment

The cost of CMD treatment was a significant concern. Some participants who lacked funds to purchase medicines resorted to skipping taking their medications until they could afford them as illustrated by a partner of a patient who attends the private rural community hospital.

My wife get medication right here at [the private rural community hospital] … For hypertension, people are told to go on a certain date, but when going, they have to carry money with them, 500 Kwacha [about 50 US cents] …. For others, it may be that it is the end of the month, and their medication is finished, but because they do not have money, they do not go to the hospital because they know that even if they go, they will not receive medication…. 500 Kwacha may seem like a small amount, but it is not a small amount for us that live in the village. For HIV, medication is free (Couple 03 – Male partner of a patient with HIV & hypertension. Rural resident)

Some participants experienced a deterioration of their health condition due to skipping medications because of a lack of funds to purchase medicine.

So, finding a facility where we can get free medicine is difficult. If we do not have money, my husband could stay one or two weeks [without taking diabetes medicine] while we are looking for money. And you can only stay for a few days, maybe five to six days, without taking medicine, and then the symptoms resurface. He could get blurred sight and could not walk a long distance (Couple 10 – Female partner of a patient with HIV and diabetes. Rural resident).

In the context of diabetes management, some participants found laboratory tests prohibitively costly and, consequently, chose to avoid seeking CMD care at healthcare facilities that mandated specific laboratory tests as part of their diabetes management plan. Instead, they decided to forgo these essential tests and purchase medicine from private pharmacies to manage the costs associated with CMD care. This was mentioned by a participant who gets care at the private rural community hospital.

When I come here to [the private rural community hospital], they say that these pills are for sale and for them to conduct a diabetes test on you, they charge you 2000 Kwacha [about 2 US dollars]. If you do not have the money, they ask you to leave …The thing is the amount of money they charge. That is the problem. When you go [to the hospital], they tell you, “Let us do this test to diagnose you.” Then, they are unable to diagnose you and say, “Let us test you again.” That is 4000 Kwacha [about 4 US dollars] already. Maybe they conduct several tests two or three times. Seeing that you do not have 4000, 6000 Kwacha, you just go straight to buy the medicine from pharmacies (Couple 07 - Male patient with HIV, diabetes, and hypertension. Rural resident)

##### Opportunity costs related to healthcare visits and long wait times at health facilities

Participants, specifically those with diabetes, expressed concerns about the long amounts of time they had to spend in the health facilities before finally receiving care. Moreover, because participants living in this region often perform piecework, a form of temporary employment such as day labor, a full day at the clinic can result in a major loss of wages, creating financial setbacks and further reducing the incentive to seek care. Some participants emphasized that the long wait times to accessing diabetes care were not experienced when seeking HIV care services as illustrated by the partner of a couple that receives health care at the tertiary government hospital.

If I had come for HIV services in the morning, I could have gone back home [early]. But if he [the partner] comes for diabetes treatment in the morning, he will get treatment at 2 or 3 pm. Sometimes, even at 4 pm (Couple 20 – Female partner of a patient with HIV and diabetes. Rural resident).

Some participants talked about experiences of making repeated visits to health facilities in a bid to access CMD medications, which were commonly out of stock in some health facilities. This issue disproportionately impacted those who heavily relied on the government health facilities to access their medications because they could not afford the cost of these medications at local pharmacies or private health facilities.

When she does come to the [tertiary government hospital] and finds that [antihypertensive medications] are not available, she comes back to the hospital another day, like maybe after two days. She makes repeated visits to the clinic because when they do tell her to buy [from private clinics], the medication is really expensive, and it starts from 5000 Kwacha [about 5 US dollars] at private clinics. So, she finds it difficult to pay 5000 Kwacha, 10,000 Kwacha for such little medication (Couple 12 – Male partner of a patient with HIV and hypertension. Urban resident)

#### Barriers related to appropriateness and ability to engage

This theme relates to the suitability of care provided by health facilities, including the quality and timeliness of the service and how involved patients are in managing their health [24].

##### Inadequate monitoring of health conditions

One of the challenges experienced by some of those receiving CMD care in government health facilities is the noncomprehensive monitoring of their progress. Some participants reported instances during follow-up appointments in which the health care providers performed some health assessments, such as measuring patients’ weight, but not others. For example, one participant who received health care at the tertiary government hospital stated that sometimes patients did not have their blood pressure (BP) checked because of unavailable or non-functioning BP machines.

Sometimes at the hypertension treatment station at the clinic, you would get weighed to see if you are gaining weight or not, but when it came to checking the blood pressure, sometimes they would say, “The machine is not working.” So, you just get the [antiretroviral medicine] and go home without having the blood pressure checked (Couple 13 – Female patient with HIV and hypertension. Rural resident).

In some cases, the lack of BP measurement may interfere with the course of treatment as alluded to by one of the participants attending care at the tertiary government hospital.

I remember some time back, the machine they use to check our blood pressure at the hospital… I do not know what had happened. Maybe it had developed some faults, but we were not being checked… but then they could not give medicine without checking the blood pressure (Couple 25 – Male patient with HIV and hypertension. Urban resident)

When functioning BP measurement machines were unavailable at government health facilities, some participants who could afford CMD care elsewhere chose to seek healthcare at better-equipped facilities, such as mission or private hospitals, where BP monitoring was more reliable and consistent, as highlighted by a participant who attends the tertiary government hospital for care.

I have been coming here for two months, and we find that the [BP measurement] machine is not working. Because hypertension is seen on a BP machine, the doctor should [use a BP machine] to see if you have high or low blood pressure. But here, sometimes, where they check your weight and BP, they say the BP machine has malfunctioned. So, when you notice the symptoms of high BP [and] you have money, you just go to a mission hospital or private hospital where they can check your BP (Couple 13 – Female patient with HIV and hypertension. Rural resident).

##### Knowledge about CMD and its management

Another challenge noted among some participants was limited knowledge of some aspects of CMD management that could negatively affect their ability to effectively engage in their own health care. All participants with CMD could accurately outline some non-pharmacological strategies for managing CMD. They were also able to provide details of the medications they were taking to manage CMD, specifically, the indication of the medication, the number of medications, the dose (in terms of number of tablets), timing, and frequency of intake. However, many had difficulties stating the specific names of the medications they were taking, and most identified their medications by size and color.

They give me aspirin; I take a quarter in the morning, the other quarter in the afternoon, and the other quarter in the evening. And there is HCTZ [hydrochlorothiazide] I take once in the morning. I do not know the name of the other one, but I also take one tablet in the morning (Couple 03 – Female patient with HIV and hypertension. Rural resident)

One of the possible contributors to the limited knowledge about the names of the prescribed medications was insufficient communication from healthcare providers about medication names, as highlighted by one of the participants who receives health care at the tertiary government hospital.

They give us [antihypertensives)…. I do not know [their] name, but they are small yellow tablets… they [healthcare providers] have never told us their name (Couple 22 – Male patient with HIV and hypertension. Rural resident)

Apart from getting limited information regarding medication names, some stated that they were provided minimal advice on various strategies to manage their health condition.

They [healthcare providers] did not give me any advice, even though it was my first time…. they did not give me any advice. They just said, “You should be [taking] these [medications daily] because the blood pressure is high. You should be [taking] them twice a day”. That is where we left each other (Couple 22 - Male patient with HIV and hypertension. Rural resident who receives health care at the tertiary government hospital).

Another factor that could negatively impact the capacity of individuals with CMD to manage their health effectively is low literacy levels (education levels shown in Table 1). Some participants highlighted a challenge that people with low education levels might have in deciphering written health-related instructions, such as follow-up appointment dates, which necessitated the need to seek assistance from family members, including spouses, to help read and interpret the written health information.

When they (healthcare providers) tell me to come [to a health facility] on such and such a month, I also tell [my husband] since I do not read. So, I take my [health record] book and give him [to] check my appointment date (Couple 09 – Female partner with HIV and hypertension. Rural resident)

### Role of partners in helping mitigate barriers to CMD care access

The partners of individuals living with HIV and CMD provided various forms of support to help mitigate barriers related to accessing CMD care. The types of support partners provided are organized based on demand-side dimensions of Levesque’s conceptual framework, i.e., how the partners supported patients’ ability to perceive, seek, reach, pay, and engage [24].

#### Partner support: ability to perceive the need for health care and seek the appropriate care

In the decision-making process regarding the need for CMD care and seeking the appropriate care, partners played a role in helping their partners opt for healthcare services instead of turning to traditional healers for help.

I tell her that a traditional healer is a person like us. Let us concentrate on the hospital. Because the traditional healer will cheat us, he will tell us that it is witchcraft, “Your neighbors are bewitching you.” I told her that we should not waste time with the traditional healers but go to the hospital (Couple 04 - Male partner of a patient with HIV and hypertension. Partner has diabetes. Rural resident)

In certain instances where patients initially sought help from traditional healers and found no improvement, their partners played a crucial role in guiding them toward medical care. These partners assisted in decision-making and ensured access to medical help, as illustrated by one participant who, after seeking help from a traditional healer with no improvement in symptoms, discussed the matter with their partner. The partner then took the initiative to ensure the participant accessed medical care.

I had frequent urination… I could not see properly. The traditional healer just gave me roots. They did not help me… When I explained to my wife after the traditional healer failed to help us, she took me to the clinic. When we got there, she said I should get tested for diabetes. She was the one leading. She did not show disappointment. She just accepted the situation (Couple 07 - Male patient with HIV, diabetes, and hypertension. Rural resident)

#### Partner support: ability to reach and pay for CMD care services

One of the main forms of support partners provided was help with transportation and medication expenses. The nature of this assistance seemed to be informed by gender norms, with male partners commonly providing financial assistance. A female participant expressed that she expected her husband to offer financial help.

If my medication for hypertension is finished and I do not have money to come to the hospital to get the medication, then it means I cannot come [to the hospital]. So, he is supposed to help me [with money] (Couple 16 - Female patient with HIV and hypertension. Rural resident)

Male partners also considered it their responsibility to resolve any financial obstacles their partners might face in accessing health care. They provided financial support to cover transportation and treatment expenses.

She tells me about every problem, if it needs money, I try to find it so that the problem can be solved…The time she was not feeling well, … I tried to find transport [money] so that she would not fail to come to the hospital to get medication and check her condition (Couple 04 - Male partner of a patient with HIV and hypertension. Rural resident)

In certain instances, the male partner needed CMD care, but the couple lacked the funds to access health care. In one such situation, the female partner offered support by seeking help from neighbors and other family members.

When I do not have anything, I do not let that disturb us from coming to the hospital. So, I beg for help from the neighbor. I can also call our sisters and tell them we have been admitted to a hospital (Couple 07 - Female partner of a patient with HIV, hypertension and diabetes. Rural resident)

Despite couples employing various strategies to address affordability and availability issues, there were instances where medication shortages at government health facilities or the considerable distances to these facilities, coupled with the inability to afford purchasing medication from nearby non-government health facilities, led to the postponement of treatment until funds became available. This challenge is illustrated by Couple 10, who receive free HIV healthcare from the private rural community hospital. However, as the partner indicates, affording diabetes treatment presents a major challenge.

I worry much over DM compared to HIV because since I started taking the ARVs, I don’t see any problem in my family or even health wise, we don’t get sick often. But this diabetes disease is what I see that it poses much threat because the medicine could run out and we have no money to buy as the government facilities are far away…. Here at [the private rural community hospital] we are able to buy [the medicine]. You can buy for the whole month, even two, if you have the money…. If you don’t have money, it means he could stay one or two weeks while you are looking for money and you will only stay for a few days without taking medicine, then that symptoms resurface…. So, I see that diabetes is very dangerous…. For HIV, we just come to the hospital to get [medicine]. We only look for transport from home to hospital, but the treatment is free (Couple 10 - Female partner of a patient with HIV and diabetes. Rural resident)

#### Partner support: ability to engage in health care

Partners played a crucial role in helping implement the patient’s health care plan. Individuals whose partners faced challenges reading or comprehending health-related communications made use of medical record documents, such as the health passport book, to assist their partners in managing their plan of care. They achieved this by interpreting the written health instructions, communicating the information to their partners, keeping track of the medical records, and providing reminders about important details like follow-up appointment dates.

My wife is unable to check [the appointment dates]. I am the one who does. When she returns from the hospital, she is supposed to give me her health passport so I can see what they have written. I then tell her what they have written and the date that she should go to the hospital. I remind her on that day, and she goes [to the hospital] (Couple 04 - Male partner of a patient with HIV and hypertension. Rural resident)

The majority of partners offered support by assisting with medication adherence.

About hypertension, she reminds me to take medicine. Last week, I was working on something, and she asked me if I had taken the medicine, and I said no. She then insisted I take the medicine right away (Couple 23 - Male patient with HIV and hypertension. Rural resident)

In addition to helping with medication adherence, some partners helped support healthy lifestyle practices, often in highly gendered ways. For example, men provided funds to buy the recommended food items, women helped prepare healthy meals, and men and women encouraged their partners to follow the recommended dietary modifications, such as reducing salt intake.

For blood sugar levels to normalize, I help in terms of diet by giving him the recommended food and ensuring he is properly taking his medications (Couple 21 – Female partner of patient with HIV and diabetes. Urban resident)

## Discussion

The research findings highlight barriers to accessing CMD care among people living with HIV in Malawi. We also identified the ways that partners helped mitigate these barriers. The findings were framed within Levesque’s health care access conceptual framework [24]. The main barriers to CMD care access included shortage of medications, cost of tests and treatments, high cost of transportation to health facilities, lengthy wait times at health facilities, faulty or unavailable medical equipment and supplies, inadequate monitoring of patients’ health condition, traditional beliefs about causes of illness, preference for herbal medicines over prescribed medicine, and inadequate knowledge about CMD treatments.

Consistent with previous studies, the commonly cited barrier to CMD care was the availability of CMD medications [12, 21, 44, 45]. Frequent CMD medication shortages at government health facilities prompted patients to purchase medications at private pharmacies, which not everyone could afford. In contrast, ART was readily available and free at the health facilities. Frequent medication shortages led to various challenges, including navigating multiple health facilities to access care, incurring additional costs of purchasing medications from private facilities, forgoing CMD treatment due to financial constraints, and resorting to alternative therapies. Some participants perceived CMD as challenging and even dangerous due to difficulties accessing CMD medications. Our findings indicate that there may be better availability of CMD medications at private health facilities but the cost of accessing CMD treatment in such a facility remains prohibitive for some community members. Additionally, diabetes medication availability appeared to be better at the tertiary government hospital. However, the distance and transportation expenses associated with seeking care at such a facility posed additional challenges for patients residing far from them. These findings underscore the need for targeted interventions to reduce financial barriers and ensure equitable access to CMD treatment.

Considering that HIV care is better funded than CMD care in Malawi, optimizing the efficiency of the limited healthcare resources could be achieved by leveraging the existing HIV care infrastructure to deliver CMD care services [46, 47]. Results from certain sub-Saharan countries, namely Uganda and Tanzania, indicate that integrating HIV and CMD care improves patient retention rates, diabetes and hypertension control, and viral suppression rates [48]. Even though integration of HIV and CMD care services could be feasible in Malawi, persistent challenges such as transportation costs and CMD medication stockouts remain [17, 45]. These challenges call for urgent innovative approaches to improve equitable access to CMD medications. Examples of such interventions include a community-based medication delivery system in western Kenya for patients with hypertension, where, in between hospital visits, pharmacists deliver the prescribed medications to patients at conveniently located drop-off points within the community. This intervention significantly improved medication adherence and BP control [49]. Another measure also implemented in rural western Kenya is the revolving fund pharmacies, where funds generated from medication sales serve as an emergency backup plan to replenish pharmacy stocks in the event of medication shortages [50]. Decentralizing integrated HIV and CMD care to the community level and incorporating it with microfinance activities has the potential not only to enhance access to CMD care but also to strengthen the financial capacity of community members who are disproportionately affected by inequalities in CMD care access due to poverty [51].

The first step of health care access is ensuring that individuals who need health care can perceive the necessity of those services and are well informed about how to access them [24]. Our findings reveal that even though PLWH and their partners may clearly understand that CMD care is available at healthcare facilities, some cultural beliefs (e.g., attributing symptoms of illness to being bewitched) may delay access to conventional health care. The attribution of CMD symptoms to bewitchment has previously been observed in other populations in Malawi, Ghana, and Nigeria [52–54]. Notably, in the present study, only a few participants sought help from a traditional healer, with those initially seeking assistance from a traditional healer transitioning to seeking care from health facilities as symptoms worsened. These findings highlight the impact of cultural beliefs on health-seeking behavior and underscore the importance of health education initiatives that address cultural healing beliefs and care-seeking trajectories.

Our research findings on the acceptability of health care and the ability to seek medical care for CMD show that many PLWH incorporated herbs such as hibiscus, ginger, and lemon grass in managing CMD. These findings align with previous studies within and outside Africa, indicating a widespread use of herbal products to manage hypertension and diabetes [21, 55–59]. In our study, the majority of individuals living with HIV and CMD used herbal therapies as adjuncts to prescribed medicine. However, some individuals had initially opted for herbal therapies as an alternative to biomedicine, delaying their access to CMD care. Since the efficacy and safety of many herbs in CMD management are not yet established [58, 60], health care providers should assess for and discuss their usage with patients as part of the shared decision-making process [61].

The present study findings show that concerns about the long-term use of CMD medications, coupled with misinformation and fears of the medications’ adverse effects, are major factors that can influence individuals’ choices and ultimately delay care-seeking behavior. We noted that local media, particularly radio broadcasts promoting other therapies, and community members’ health-related advice, played a role in shaping decision-making about where to seek care. Overall, these findings emphasize the need for targeted communication and health education at individual, couple/family, and community levels that address misconceptions about CMD management and promote informed decision-making in seeking care for CMD. Since several participants mentioned receiving information about CMD through radio, using the radio may be a valuable strategy to enhance awareness of CMD and its prevention and management.

Our research findings show that PLWH faced several challenges related to the appropriateness of the health care provided and the ability to engage in CMD care. Inadequate monitoring of patients’ progress due to the scarcity of functioning equipment, specifically BP monitoring machines, was a major challenge that has also been noted in previous studies in Malawi and Uganda [62–64]. Addressing the availability and functionality of essential monitoring equipment for CMD in healthcare facilities will improve the quality of care and, ultimately, improve health outcomes for PLWH. Most participants could not articulate the names of the CMD medications they were taking and mostly identified medications by size and color. These findings are consistent with previous studies conducted in outpatient clinics in Ghana, Ethiopia, and Sri Lanka, revealing that many patients have difficulty identifying their medications by name [65–67]. Studies show that knowledge about prescribed medications is positively associated with medication adherence [68] and better disease control [69]. User-friendly written information on the prescribed medications, in addition to verbal instructions, can help improve medication-related health literacy [67, 70].

Our study findings highlight the multifaceted support that partners provide to help mitigate barriers to accessing CMD care. In the ability to perceive the need for CMD care and seek appropriate health care, partners actively engaged in the decision-making process with their significant others. In cases where PLWH and CMD had initially sought help from traditional healers, collaborative decision-making with their partners helped redirect them toward conventional health care. These findings point to the important role of partners in health-related decision-making and their potential role in facilitating timely access to health care [71, 72].

Partners of PLWH and CMD played a crucial role in mitigating barriers related to the ability to reach and pay for CMD care services. Our study revealed a gendered pattern in the support provided, with male partners commonly offering financial help with transport and treatment expenses. This points to traditional gender relations characterized by clear divisions of labor, where men are expected to fulfill the role of providers for the family’s financial needs [73].In instances where both partners faced financial constraints, female partners reported using strategies such as seeking financial assistance from neighbors and other family members. In addition to cultural norms, this gendered support pattern may be partly explained by the fact that in many countries worldwide, including Malawi, women have fewer financial resources than men [74, 75]. The practice of seeking help from family members and neighbors provides evidence of Ubuntu culture, also referred to as *umunthu in* Malawi, that promotes virtues such as solidarity, group support, sharing, and caring for one another [76, 77].

The research findings also demonstrate the role partners play in facilitating individuals’ engagement in health care. Particularly noteworthy is the support provided by partners in navigating health communication challenges, where those with partners facing difficulties in understanding health-related information utilized tools like health passport books to interpret and convey information to their partners. Partners also assisted in fostering healthy lifestyle practices and medication adherence, as demonstrated in other studies with couples living with HIV in Malawi and other African settings [28–30]. This multifaceted assistance highlights the comprehensive impact partners can have in enhancing the overall healthcare experience for individuals, addressing communication barriers, and promoting holistic well-being. Studies among patients with CMD have demonstrated that involving family members in the management plan improves patients’ health outcomes, including health literacy, self-efficacy, self-management, medication adherence, and disease control [78–80]. However, other findings show that factors such as interpersonal conflicts arising from constant reminders by partner to adhere to plan of care, sabotaging of lifestyle modification efforts, and intimate partner violence can negatively impact health outcomes [29, 81, 82]. This points to the importance of healthy relationship dynamics in dyadic couple management of chronic medical conditions.

## Limitations

One limitation of this study is that it was conducted in only three health facilities in the Zomba district of Malawi. Therefore, the experiences of PLWH and CMD in three health facilities might differ from the experiences of their counterparts attending other health facilities in the area or other regions in Malawi, reducing the transferability of the findings. A second limitation is that participants with diabetes were under-represented in the study sample, which may have limited the breadth of information garnered on the barriers PLWH and diabetes face in accessing CMD care and ways that their partners support them. Third, our study focused on how partners positively supported access to CMD care. Given that studies have shown that partners can negatively impact chronic disease management [29, 82], future studies should also explore how couple relationship dynamics can negatively impact access to CMD care. Despite the limitations noted, this study provides valuable insights into how couples support one another to mitigate barriers to accessing health care. Future studies should examine other family-level sources of support.

## Conclusion

Many barriers to accessing CMD care were identified for people living with HIV and CMD, and these barriers were perceived as posing greater challenges than accessing HIV care. Partners provided critical forms of support and were an important source of resilience in navigating these barriers. With the rise of CMD among people living with HIV, improving access to high-quality CMD care should be prioritized using lessons learned from HIV and integrated care approaches. Partners should be involved in CMD care to enhance the ability of PLWH to access the appropriate healthcare services, which is paramount in settings with poor healthcare infrastructure for noncommunicable diseases.

## Data Availability

Data are available upon reasonable request from the corresponding author.

## Abbreviations

ART: Antiretroviral therapy
BP: Blood pressure
CMD: Cardiometabolic disorders
CVD: Cardiovascular disease
DM: Diabetes mellitus
DALYs: disability-adjusted life-years
HIV: Human immunodeficiency virus
HTN: Hypertension
PLWH: People living with HIV
SSA: Sub-Saharan Africa
UNAIDS: Joint United Nations Programme on HIV/AIDS
